# Study of Factors Influencing Moisture Susceptibility of Warm-Mix Asphalt Using the Surface Free Energy Approach

**DOI:** 10.3390/polym15132798

**Published:** 2023-06-23

**Authors:** Liping Liu, Lingxiao Liu, Ying Yu

**Affiliations:** The Key Laboratory of Road and Traffic Engineering, Ministry of Education, Tongji University, Shanghai 201804, China

**Keywords:** warm-mix asphalt, moisture susceptibility, surface free energy (SFE), three-phase model, effective adhesion work

## Abstract

The application of warm-mixing technology brings considerable economical and environment benefits by decreasing the mixing temperature during warm asphalt mixture (WMA) production. However, the possible water residue also generates concerns for moisture susceptibility. For deep investigation on the influencing factors and mechanisms of the moisture susceptibility of WMA, surface free energy (SFE) tests and laboratory tests are applied in this research. A novel indicator based on SFE, namely, effective adhesion work, is proposed to assess the asphalt–aggregate adhesion with different moisture contents. Then, given the mixing procedure of the dry-mixing method, an advanced three-phase model as a form of asphalt–aggregate-warm mixing additive is introduced, improving the conventional two-phase asphalt–aggregate model for better reflecting the separate addition of warm-mixing additives during mixing. Afterwards, the influence of aggregate type, asphalt type, aggregate moisture content, warm-mixing agent type, and the warm-mixing process on the moisture susceptibility of WMA is analyzed utilizing the models and indicators proposed. Finally, the validity of the SFE indicator is verified by comparing the calculation of effective adhesion work with freeze–thaw splitting test results. The results show that all of the above factors impact the moisture susceptibility of WMA by influencing the interfacial adhesion, with the effect of moisture content being the most significant. Meanwhile, effective adhesion work and the three-phase model brought out in this research are proven to be feasible to characterize the adhesion properties of WMA, offering theoretical support to the research on warm-mixing technology.

## 1. Introduction

Warm-mixing technology allows asphalt to reach the viscosity needed for mixing at lower temperatures and, therefore, is able to reduce the mixing temperature by 30–40 °C during the asphalt mixture production, resulting in less energy consumption, better construction convenience, and less aging of the asphalt [1,2,3,4,5]. However, problems also arise in that the moisture susceptibility may deteriorate as a consequence of lower mixing and compacting temperatures [6,7,8].

Relative studies have pointed out that the moisture damage of a mixture is attributed to the adhesion failure between asphalt and aggregate and, therefore, researchers have introduced various methods and indicators to characterize the adhesion properties [9,10,11,12]. Currently, many methods haven been brought out to estimate the moisture susceptibility of asphalt mixtures. For instance, the boiling method indicates the moisture susceptibility by comparing the stripping of the mixed aggregates before and after suffering boiling, but the process takes a long time and is subjective [13]. Photoelectric colorimetry uses the relationship between the concentration of a substance at a determined wavelength and the absorption effect of light to determine the absorbance of a solution and then calculate the concentration, but the operation is very complex [14]. Atomic force microscopy (AFM) is able to characterize the adhesive property between asphalt and aggregate in nanoscale, but the sample preparation is demanding [15]. As for surface free energy (SFE), there is no need for specimen compaction and the adhesive between the asphalt and aggregates can be simply calculated by testing the SFE parameters. Therefore, SFE obtains more attention in present studies on the moisture resistance of asphalt mixtures [16].

Elphingstone introduced SFE to asphalt mixture research for the first time to study the interfacial cracking prediction in hot-mix asphalt (HMA) mixtures [17]. Cheng measured the SFE indexes of different asphalts and aggregates and calculated the cohesion work of asphalt and the adhesion work of the asphalt–aggregate interface. A comparison between the SFE test and conventional moisture susceptibility tests confirmed the feasibility of SFE indicators to evaluate the moisture susceptibility [18]. Zhang et al. compared the SFE test result with adhesion grade and TSR obtained from a laboratory test of six different WMAs, and the relevance among them was studied [19]. Xiao et al. utilized an SFE test on asphalt with different aging degrees and found that the aging process had an adverse influence on moisture susceptibility based on SFE indicators, but also pointed out the limitation of the SFE method in characterizing the impact of the penetrating of asphalt into pores on aggregates [20]. Alvarez et al. utilized SFE to determine the optimal dose of WMA additives for achieving the best asphalt–aggregate adhesion [21]. Ali et al. combined both SFE and laboratory tests to investigate the effect of additives as well as aging on the moisture resistance of asphalt mixtures [22]. Yang et al. analyzed the mechanism of a certain warm-mix additive using an SFE test [23]. Habal et al. compared the water resistance of a mixture with different warm-mix additives and aggregate–asphalt combinations using SFE and laboratory tests, finding a good relationship between the SFE indicators and performance test results. Furthermore, a threshold was established based on SFE whose accuracy is a favorable 90% [24,25].

There are more factors impacting the moisture susceptibility of WMA compared with HMA [7]. In addition to the commonly accepted factors of HMA, the involvement of warm-mixing additives and the mixing procedure applied also attracted the attention of researchers in terms of their impact on the moisture susceptibility of WMA. Hurley et al. found that the influence of different warm-mixing additives on different aggregates is distinct [26,27,28]. Zaumanis noted that the poor adhesion between asphalt and aggregates may occur due to unevaporated water remaining during some warm-mixing process, thus leading to the negative performance of WMA [29].

Current research has carried out remarkable investigations into SFE theory and the influencing factors on the moisture susceptibility of WMA. It can still be noticed that few studies concern the influence of moisture content and the mixing process and are therefore unable to precisely estimate the moisture susceptibility of WMA, especially with dry-mix additives. In this research, on the basis of SFE theory, a novel indicator for evaluating the asphalt–aggregate adhesive property with different moisture contents is proposed. Moreover, a three-phase model of aggregate–asphalt-warm mixing additives is introduced and enhanced from the conventional two-phase model by taking the process of dry mixing into consideration. The influence of several factors on the moisture susceptibility is analyzed using the advanced indicator and model put forward in this paper and the freeze–thaw splitting test is applied for the verification of the SFE test.

## 2. Surface Free Energy Theory

SFE theory provides a quantitative measurement method for adhesion properties between aggregates and asphalt. Understanding the SFE components of asphalts and aggregates contributes to the prediction of moisture susceptibility.

### 2.1. Two-Phase Model

In the traditional two-phase model, the adhesion process of asphalt and aggregate can be expressed as asphalt + stone → asphalt–stone. The amount of energy change per unit area of the adhesion interface is the adhesion work (W_as_). The larger the W_as_ is, the stronger the asphalt–stone interface is. The adhesion work without moisture can be calculated according to Equation (1). When water-related damage appears in the asphalt pavement, water enters the void, and then gradually adheres to the aggregate by replacing the asphalt. This process requires work by external forces, which is called the adhesion work with moisture (W_asw_), the physical meaning of which is the energy change per unit after two contacted materials are separated by water. The larger the W_asw_ is, the weaker the asphalt–stone interface is. The adhesion work with moisture can be calculated according to Equation (2) [30].

(1) $$W_{\mathrm{as}}=\gamma_{a}+\gamma_{s}-\gamma_{\mathrm{as}}=2\sqrt{\gamma_{s}^{LW}\gamma_{a}^{LW}}+2\sqrt{\gamma_{s}^{+}\gamma_{a}^{-}}+2\sqrt{\gamma_{s}^{-}\gamma_{a}^{+}}$$

(2) $$W_{\mathrm{asw}}=\gamma_{sw}+\gamma_{aw}-\gamma_{as}=2\gamma_{w}+2\sqrt{\gamma_{a}^{LW}\gamma_{s}^{LW}}+2\sqrt{\gamma_{a}^{+}\gamma_{s}^{-}}+2\sqrt{\gamma_{a}^{-}\gamma_{s}^{+}}-2\sqrt{\gamma_{a}^{LW}\gamma_{w}^{LW}}\;-2\sqrt{\gamma_{s}^{LW}\gamma_{w}^{LW}}-2\sqrt{\gamma_{a}^{+}\gamma_{w}^{-}}-2\sqrt{\gamma_{a}^{-}\gamma_{w}^{+}}-2\sqrt{\gamma_{s}^{+}\gamma_{w}^{-}}-2\sqrt{\gamma_{s}^{-}\gamma_{w}^{+}}$$

where $\gamma_{a}$, $\gamma_{s}$, and $\gamma_{w}$ represent the surface free energy of asphalt, stone, and water, respectively, mJ·m^−2^; $\gamma_{sw}$,$\gamma_{as}$, and $\gamma_{aw}$ represent the stone–water, asphalt-stone, and asphalt–water interface energy, mJ·m^−2^; $\gamma^{LW}$ is the van der Waals component, mJ·m^−2^; and $\gamma^{+}$ and $\gamma^{-}$ are the Lewis acid term and base term, mJ·m^−2^.

In addition, some researchers have proposed some comprehensive indicators by considering the adhesion work with/without moisture and cohesion work of asphalt, such as ER_1_, ER_2_, ER_1_·SSA, ER_2_·SSA, etc. Among them, ER_2_ is proved to be well-correlated with indicators of moisture susceptibility, and furthermore, a threshold value is recommended in an NCHRP report [31,32]. Therefore, in this paper, ER_2_ is used as the comprehensive indicator, noted as ER. The larger the ER value, the better the moisture susceptibility of the corresponding asphalt mixture. ER can be calculated using Equation (3).

(3) $$ER=\left|\frac{W_{as}-2\gamma_{a}}{W_{asw}}\right|$$

### 2.2. Three-Phase Adhesion Model

**Figure 1:**
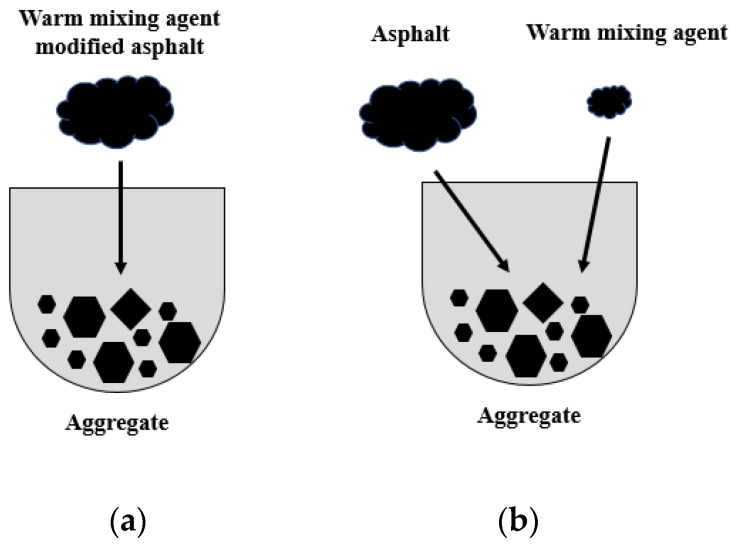
(**a**) Wet; (**b**) dry-mixing process.

In the conventional two-phase adhesion model, only asphalt (or warm-mix additive modified asphalt) and aggregate are considered, as shown in Figure 1a, which is suitable for wet-mixing processes. However, in the engineering practice of WMA, dry mixing is also widely used, during which the aggregates are first mixed with warm-mix additives and afterwards with asphalt, as shown in Figure 1b. This process is absolutely inconsistent with the original two-phase model. Hence, a corresponding three-phase model needs to be established which takes the warm-mix agent into account. The adhesion in the dry-mixing method can be expressed as asphalt + extra agent + stone → asphalt-extra agent-stone. According to its energy change, the corresponding formula of adhesion work without moisture (W_ase_) can be introduced, as shown in Equation (4).

(4) $$\begin{array}{ll} w_{\mathrm{ase}} & =\gamma_{\mathrm{ae}}+\gamma_{se}-\gamma_{a}-2\gamma_{e}-\gamma_{s}\\ & =2(\sqrt{\gamma_{e}^{LW}\gamma_{a}^{LW}}+\sqrt{\gamma_{s}^{LW}\gamma_{e}^{LW}}+\sqrt{\gamma_{e}^{+}\gamma_{a}^{-}}+\sqrt{\gamma_{e}^{-}\gamma_{a}^{+}}+\sqrt{\gamma_{e}^{+}\gamma_{s}^{-}}+\sqrt{\gamma_{e}^{-}\gamma_{s}^{+}}) \end{array}$$

where γ_ae_ and γ_se_ denote the interfacial energy of the asphalt-extra agent and aggregate-extra agent, respectively, mJ·m^−2^; $\gamma_{e}$ is the surface free energy of the extra agent.

**Figure 2:**
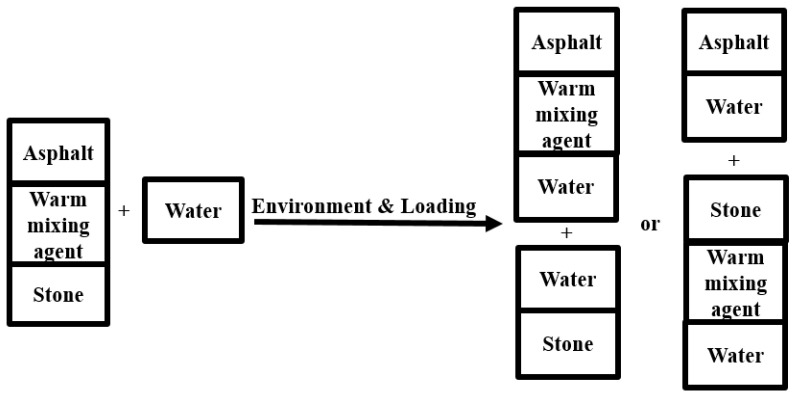
The adhesion failure process with moisture in dry mixing.

The adhesion failure process with moisture of the three-phase model is complex. The interfacial failure caused by water may occur at two interfaces. One is the warm-mixing agent–asphalt interface, and the other is the warm-mixing agent–aggregate interface, as shown in Figure 2. Assuming a 50/50 split between the two scenarios, the formula of adhesion with moisture is calculated as Equation (5), and ER can be calculated as Equation (6).

(5) $$\begin{array}{ll} w_{\mathrm{asew}} & =\frac{1}{2}(2\gamma_{ew}+2\gamma_{sw}-\gamma_{ae}-\gamma_{se})\\ & =\sqrt{\gamma_{a}^{LW}\gamma_{e}^{LW}}+\sqrt{\gamma_{e}^{LW}\gamma_{s}^{LW}}+\sqrt{\gamma_{a}^{+}\gamma_{e}^{-}}+\sqrt{\gamma_{a}^{-}\gamma_{e}^{+}}+\sqrt{\gamma_{e}^{+}\gamma_{s}^{-}}\;+\sqrt{\gamma_{e}^{-}\gamma_{s}^{+}}\;-2\sqrt{\gamma_{e}^{LW}\gamma_{w}^{LW}}-2\sqrt{\gamma_{s}^{LW}\gamma_{w}^{LW}}\;-2\sqrt{\gamma_{e}^{+}\gamma_{w}^{-}}\;-2\sqrt{\gamma_{e}^{-}\gamma_{w}^{+}}-2\sqrt{\gamma_{s}^{+}\gamma_{w}^{-}}-2\sqrt{\gamma_{s}^{-}\gamma_{w}^{+}}+4\gamma_{w}+\gamma_{s}-\gamma_{a} \end{array}$$

(6) $$ER=\frac{1}{2}\left|\frac{W_{as}-2\gamma_{a}}{W_{asw}}+\frac{W_{as}-2\gamma_{a}}{W_{asw}}\right|=\left|\frac{W_{as}-\gamma_{a}-\gamma_{e}}{W_{asw}}\right|$$

The three-phase model can characterize the process of the dry-mixing method and the corresponding adhesion indicators can be calculated. It enables the SFE theory to study the effect of the mixing process on the water stability of WMA.

## 3. Materials and Methods

### 3.1. Materials

In this research, two kinds of asphalt were involved, including base asphalt with penetration 60/80 and I-D linear SBS modified asphalt. The indexes of both asphalts met the requirement of the *Technical Specification for Construction of Highway Asphalt Pavements standards*, a Chinese standard [33]. The specific properties are shown in Table 1.

**Figure 3:**
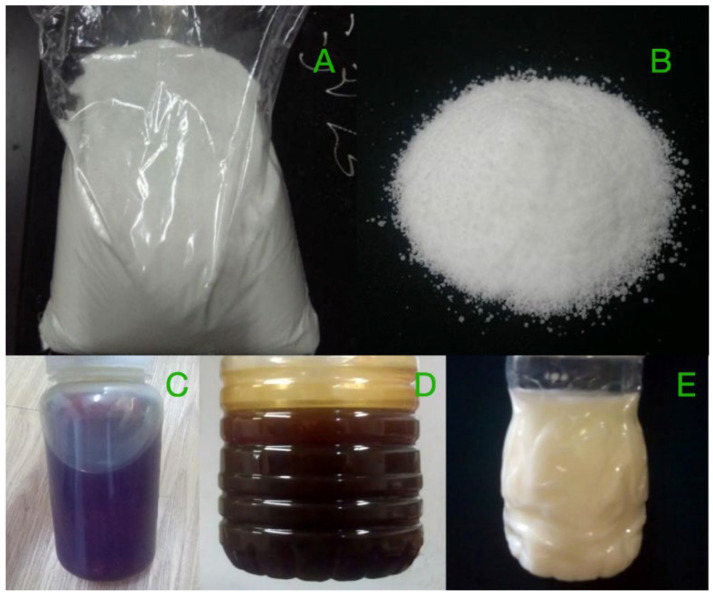
Five kinds of warm-mixing agents.

Additionally, five kinds of warm-mixing agents, shown in Figure 3, were employed in this study. Among them, agent A was surface active additive and was mixed with aggregates first and asphalt thereafter. Agent B was organic viscosity-reducing additive. Agents C and D were both brown viscous liquids. B, C, and D were all required to be mixed with asphalt first to prepare modified asphalt. Agent E was white water-soluble latex and was sprayed into a mixing pot with asphalt during mixing. The mixing content is summarized in Table 2.

Three kinds of aggregates, including limestone, basalt, and granite, which are commonly used in the field of road engineering, were selected for further research. The densities indexed are shown in Table 3.

**Figure 4:**
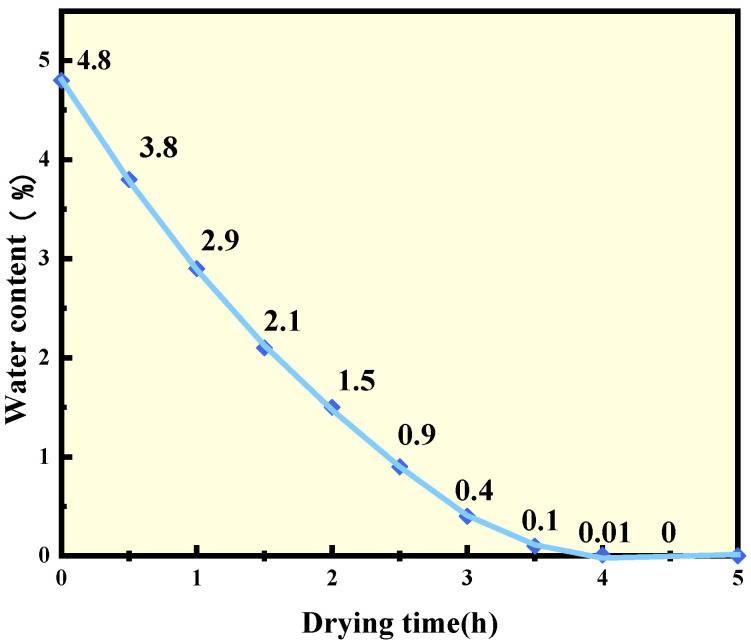
The moisture content of limestone at different drying times.

In order to study the factor of aggregate moisture content, limestone with different moisture contents was obtained by soaking limestone in water for three hours and placing it in a 145 °C oven for different times. The quality was recorded every half an hour and, therefore, the relationship between the moisture content of the aggregate and the drying time can be acknowledged, which is shown in Table 4 and Figure 4.

### 3.2. Experimental Methods

#### 3.2.1. Surface Free Energy Testing Technology

**Figure 5:**
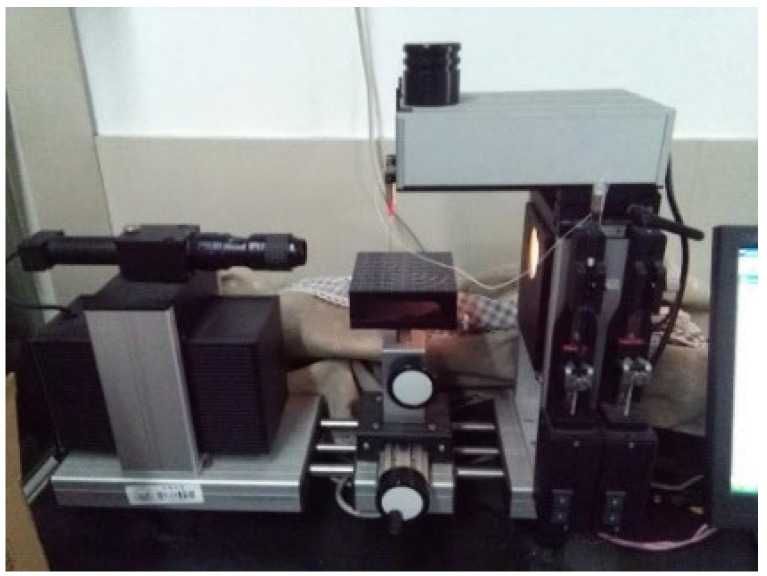
Contact angle system OCA.

**Figure 6:**
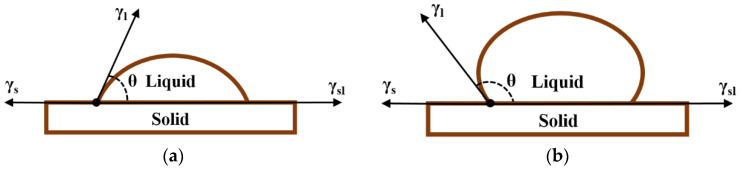
Experimental principle (**a**) θ < 90°; (**b**) θ > 90°.

The sessile drop method was employed in this research to test the surface free energy of asphalt and aggregates. The instrument used was a contact angle system OCA, as shown in Figure 5, of which the theoretical basis is the Young’s equation (Equation (7)) deduced in Figure 6. Combining it with the LW-AB model (Lewis Acid/Base Model) of Equation (8), Equation (9) was obtained. Regarding the solid as the object to be measured, by increasing the number of known liquids, the linear equation set shown in Equation (10) was established and the surface free energy parameters were obtained when the equation was solved.

(7) $$\gamma_{s}=\gamma_{l}\cos\theta +\gamma_{sl}$$

(8) $$\gamma =\gamma^{LW}+\gamma^{AB}=\gamma^{LW}+2\sqrt{\gamma^{+}\gamma^{-}}$$

(9) $$\gamma_{l}(1+\cos\theta )=2(\sqrt{\gamma_{s}^{\mathrm{LW}}\gamma_{l}^{\mathrm{LW}}}+\sqrt{\gamma_{s}^{+}\gamma_{l}^{-}}+\sqrt{\gamma_{s}^{-}\gamma_{l}^{+}})$$

(10) $$\left[\begin{array}{l} \sqrt{\gamma_{l_{1}}^{\mathrm{LW}}}\sqrt{\gamma_{l_{1}}^{+}}\sqrt{\gamma_{l_{1}}^{-}}\\ \sqrt{\gamma_{l_{2}}^{\mathrm{LW}}}\sqrt{\gamma_{l_{2}}^{+}}\sqrt{\gamma_{l_{2}}^{-}}\\ \sqrt{\gamma_{l_{3}}^{\mathrm{LW}}}\sqrt{\gamma_{l_{3}}^{+}}\sqrt{\gamma_{l_{3}}^{-}} \end{array}\right]\left[\begin{array}{l} \sqrt{\gamma_{s}^{\mathrm{LW}}}\\ \sqrt{\gamma_{s}^{-}}\\ \sqrt{\gamma_{s}^{+}} \end{array}\right]=\left[\begin{array}{l} \frac{\gamma_{l_{1}}{(1+\cos\theta }_{1})}{2}\\ \frac{\gamma_{l_{2}}{(1+\cos\theta }_{2})}{2}\\ \frac{\gamma_{l_{3}}{(1+\cos\theta }_{3})}{2} \end{array}\right]$$

where θ represents the contact angle between the test solid and known liquid; $\gamma_{s}$ and $\gamma_{l}$ represent the surface free energy of solid and liquid, respectively, mJ·m^−2^; $\gamma_{sl}$ represents the solid–liquid interface energy, mJ·m^−2^; $l_{1},l_{2},\;and\;l_{3}$ represent different known liquids.

In the NCHRP report, the surface energy parameters of five known liquids suitable for testing asphalt and aggregates were given, which are listed in Table 5 [34]. Some preliminary researches have been conducted to select proper liquids based on conditional number (CN) to reduce the impact of parameters on test results. As a consequence, distilled water, diiodomethane, and glycerin were chosen for the following test.

#### 3.2.2. Freeze–Thaw Splitting Test

According to the corresponding standard of China, the moisture susceptibility is evaluated using freeze–thaw splitting test [33,34]. The test requires two groups of four specimens prepared via 50 times of Marshall compaction in each side and one group, namely the freeze–thaw group, needs to undergo freeze–thaw conditioning, while the other group is the control group stored in ambient environment. Both groups are tested at 25 °C and the splitting strength can be obtained using Equation (11). The tensile strength ratio (TSR) can be calculated with Equation (12).

(11) $$S_{T1}\;or\;S_{T2}=\frac{0.006287P_{T}}{h}$$

(12) $$TSR=\frac{S_{T2}}{S_{T1}}\times 100$$

where TSR is tensile strength ratio (%); S_T1_ and S_T2_ are splitting tensile strength (kPa) under dry and freeze–thaw conditioning, respectively; P_T_ is the maximum load (N); and h is the height of the specimen, mm.

## 4. Results and Discussion

The factors affecting the moisture susceptibility of WMA, which are complicated, can be studied by using SFE theory. The characteristics of raw materials, the mixture design, and the mixing temperature will all make a difference [35]. In this paper, the influence of aggregate type, aggregate moisture content, warm-mix additives, asphalt type, and mixing method on the water stability of WMA are studied utilizing SFE theory.

### 4.1. Surface Free Energy Components

**Figure 7:**
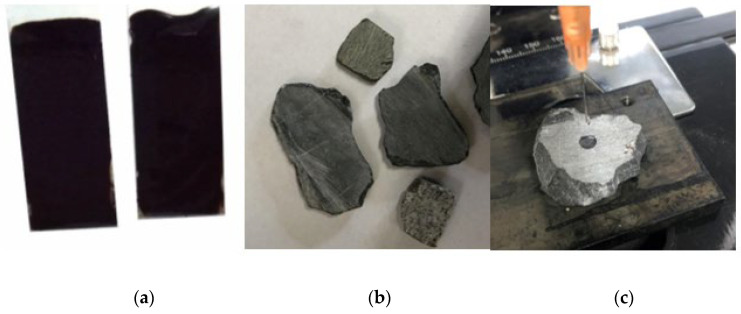
(**a**) Asphalt samples; (**b**) aggregate samples; and (**c**) testing procedure.

The test samples and the testing procedures are shown in Figure 7. The parameters based on surface free energy were calculated after contact angles were measured, and the results are shown in Table 6.

### 4.2. The Effect of Aggregate Type

**Figure 8:**
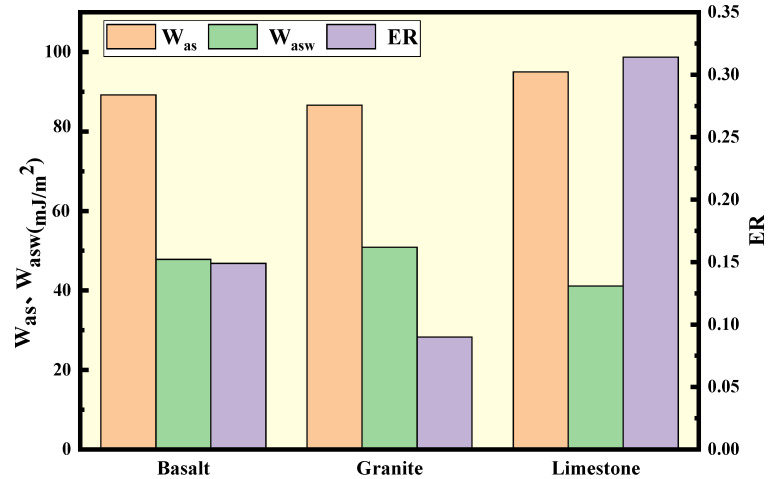
Values of adhesion indicators of different aggregates and agent A.

In order to explore the effect of aggregate type on the water stability of WMA, the adhesion parameters of basalt, granite, and limestone with agent A modified base asphalt were calculated and are shown in Figure 8. The TSR test values of corresponding mixtures were prepared and tested for verification. The mixture gradation was AC-20. Results are shown in Table 7.

It can be seen from the viewpoint of adhesion indicators that the type of aggregate had a significant influence on the asphalt–aggregate adhesion. The ranking result was limestone > basalt > granite ordered by W_as_, W_asw_, as well as ER. The ranking of TSR results shows favorable consistency with the adhesion indicators.

### 4.3. The Effect of Aggregate Moisture Content

None of the existing adhesion indicators consider the effect of aggregate moisture content. Calculation models of adhesion work, with and without moisture, respectively, simulate conditions of no water and adequate water. In this paper, the effective adhesion work is proposed based on the moisture content of aggregate in the mixtures. The physical meaning of effective adhesion is the value of the surface energy change on a unit area of the aggregate after adhesion among water, asphalt, and aggregate. The value is positively correlated with asphalt content and adhesion work without moisture, and negatively correlated with moisture content and adhesion work with moisture, as shown in Equation (13). The larger the effective adhesion work, the better the adhesion between asphalt and aggregate.

(13) $$W_{as,eff}=W_{as}\times \frac{p_{a}}{p_{a}+w}-W_{asw}\times \frac{w}{p_{a}+w}$$

where $W_{as,eff}$ is the effective adhesion work, mJ/m^2^; ω and P_a_ represent the aggregate moisture content and asphalt content, respectively, %.

The adhesion work with and without moisture between warm-mixing agent A modified asphalt and different aggregates were calculated, as shown in Table 8. Analysis of the results shows that for all combinations of asphalts and aggregates, W_asw_ was less than W_as_, meaning that water was more prone to achieve adhesion to aggregate than asphalt. This indicates that the presence of water influenced the adhesion between asphalt and aggregate, thus creating weakened adhesion areas at the interface of asphalt and aggregates, which can result in easier water invasion into the asphalt–aggregate interface and moisture damage.

**Figure 9:**
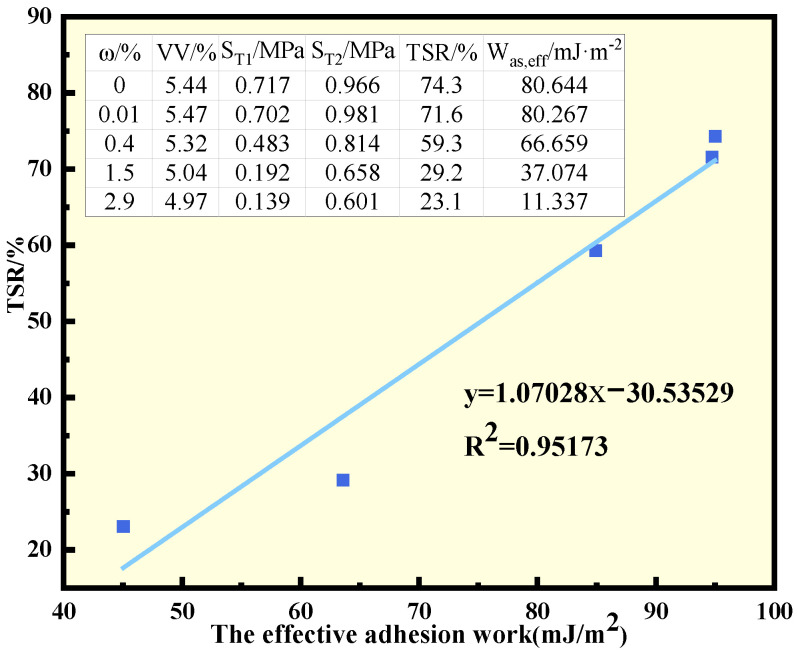
TSR, $W_{as,eff}$, and linear regression analysis.

Meanwhile, specimens using warm-mixing additive agent A and limestone with moisture contents of 0%, 0.01%, 0.4%, and 1.5%, respectively, were prepared for the freeze–thaw splitting test. The mixing temperature was 135 °C, the gradation was AC-13, and the asphalt content was 5.0%. The TSR results and effective adhesion work of mixtures with different moisture contents were summarized and then subjected to linear regression analysis, as shown in Figure 9.

From the figure, it can be seen that the effective adhesion work decreased significantly as the moisture content of aggregate increased, indicating that the presence of water in the aggregate significantly degraded the adhesion of the asphalt to the aggregate. The TSR values verified this phenomenon. As the moisture content of the aggregate increased, the splitting strength without freeze–thaw cycles decreased slightly, while that with freeze–thaw conditioning decreased sharply, leading to a dramatic decline in TSR.

Linear regression analysis shows that the correlation coefficient between TSR and W_as,eff_ calculated reached 0.95, which means strong correlation. This proves the validity of the effective adhesion work in evaluating the water stability of the WMA.

### 4.4. The Effect of Warm-Mixing Agent Type

**Figure 10:**
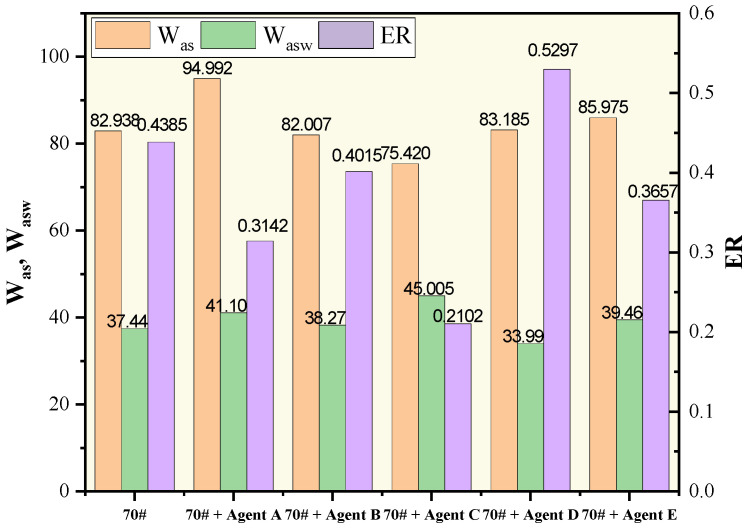
Adhesion indicators of asphalts and limestone.

It has been researched that warm-mixing agents have an important effect on the performance of WMA. In this section, the adhesion indicators of different warm-mixing modified base asphalts to limestone are calculated. The results are shown in Table 9 and Figure 10.

The ranking based on the calculation of the three adhesion indicators was different. The results ranked by the W_as_ were A > E > D > B > C, while those by the W_asw_ and ER were D > B > E > A > C. The difference emerged due to the consideration of the effect of moisture. When lacking the consideration of water, agents A and D were able to promote the adhesion as the $W_{\mathrm{as}}$ value announced, while all the agents deteriorated the adhesion when moisture was taken into account as $W_{\mathrm{asw}}$ and ER, with oil-based warm-mixing agent D having the least effect.

To verify the above findings, WMA samples using warm-mixing agents A, D, and E were compacted and the TSR test was conducted. The mixture gradation was AC-20, and the aggregate was limestone. Results are shown in Table 10.

From the view of TSR, all the warm-mixing agents degraded the moisture susceptibility, among which the mixture with agent D was the least affected. The ranking by TSR test result was consistent with the calculation of W_asw_ and ER. The reason may be the possible introduction of moisture brought by agents A and E. Agent A was a kind of water-soluble solid and, thus, can easily absorb water, while agent E was in the form of an emulsion containing water. Agent D was oil-based, and thus hydrophilic, and would not be a cause for the introduction of water. Overall, all the indicators except $W_{\mathrm{as}}$ came to the consensus that the water stability of WMA with an oil-based warm-mixing agent was better.

### 4.5. The Effect of Asphalt Type

**Figure 11:**
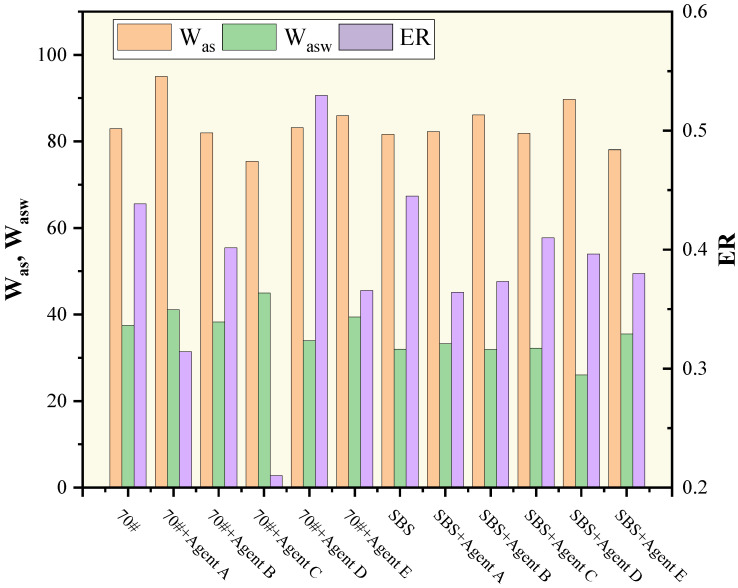
Adhesion indicators of different asphalts with different warm-mix additives.

To investigate the effect of asphalt type on the moisture stability of WMA, the adhesion indicators were calculated based on the SFE parameters of base asphalt, SBS modified asphalt, and limestone aggregates, and the result is shown in Figure 11. The freeze–thaw splitting test was conducted for verification on the hot mix and warm mix simultaneously with limestone and base asphalt or SBS modified. The warm-mix additive was agent C and the mixing temperature was 30 °C lower than that for HMA. The test result is shown in Table 11.

It can be inferred from the results that when different warm-mix additives were applied to different asphalts, the adhesion properties between asphalt and aggregate were distinct. In other words, there was compatibility between asphalt and warm-mix agents. From the calculation result of ER, agent D was the best among the five agents for base asphalt, while agent C was the best for SBS modified asphalt. The choice of asphalt can determine the application of warm-mix agents and, therefore, result in distinct performances of WMA mixtures.

In the meantime, when applying different asphalts to warm mixing, the effect on the moisture susceptibility is distinct. To take the combination of asphalt + agent C as an example, the introduction of agent C resulted in an extreme decrease in ER for base asphalt, while the ER for SBS modified asphalt was almost equivalent to that of original asphalt and was much higher than agent C modified base asphalt. This was verified by the TSR result. Also, it is worth noticing that the TSR result for the base asphalt mixture was consistent with ER value, which both showed a sharp decrease, while that of SBS modified asphalt showed a slight enhancement and was not consistent with ER. There may be other more sophisticated mechanisms for the interaction between polymer modified asphalt and warm-mix agent that compensate for the slight decline in SFE parameters.

### 4.6. The Effect of the Mixing Process

**Figure 12:**
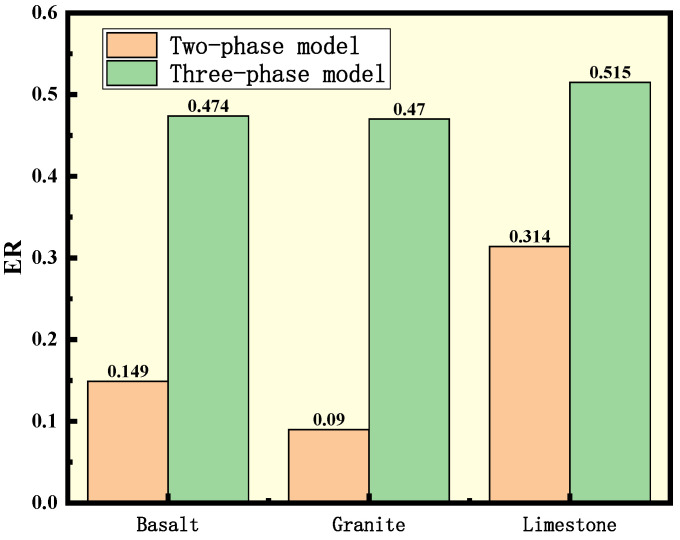
Adhesion indicators ER based on different models.

As interpreted above, the conventional two-phase asphalt–aggregate adhesion model is suitable for the wet-mixing method, in which the warm-mixing additives are added into asphalt to modified asphalt first and then mixed with aggregates. When confronted with additives needing the dry-mixing method, the adhesion of asphalt, aggregate, and warm-mixing agent should be characterized using the three-phase model proposed in previous sections. In this article, the adhesion indicator ER of agent E with base asphalt based on two- and three-phase model was calculated and is shown in Figure 12. Dry-mixing and wet-mixing WMA mixture specimens using AC-13 gradation for the TSR test were prepared with agent E, limestone, and base asphalt for validation. The mixing temperature was 135 °C. The freeze–thaw splitting test results are shown in Table 12.

For different types of aggregates, the ER values of the three-phase model were greater than those of the two-phase model, indicating better adhesion prepared using the dry-mixing method. The TSR result demonstrated the theoretical calculation, with mixtures prepared using the dry-mixing method superior to those using the wet-mixing method. This consistency also indicates that the three-phase model proposed in this paper is effective for predicting the water stability of the WMA prepared with the dry-mixing method.

## 5. Conclusions

Using surface free energy theory, the influence of several factors on the moisture susceptibility of WMA focusing on the adhesion properties of asphalt–aggregate interface was studied, and the following conclusions were drawn:

1. Aggregate type, moisture content of aggregate, warm-mixing agent type, asphalt type, and mixing process have significant effects on the water stability of WMA, and the conclusions of adhesion indicators based on SFE and conventional moisture susceptibility test method results are consistent. Specifically, the water content of the aggregate state is the most significant factor affecting the moisture susceptibility. The presence of water greatly affects the performance of the mixture. Therefore, the dryness of the aggregate should be strictly controlled in WMA.
2. Based on the surface free energy theory, the effective adhesion work considering the water content of the aggregate is proposed to characterize the aggregate–asphalt adhesion condition under different water contents. This indicator is highly correlated with TSR value and can be used as a convenient index to predict the moisture susceptibility of WMA.
3. A three-phase model of asphalt-warm mixing agent–aggregate is proposed according to the production process of the dry-mixing method. The corresponding calculation equations of adhesion indicators were also derived. The consistency between the adhesion indicator and TSR indicated that the three-phase model is applicable to the adhesion process of WMA prepared using the dry-mixing method.

## Figures and Tables

**Table 1:** Properties of asphalt.

Indicator	Penetration (25 °C, 100 g)	Ductility * (cm, 5 cm/min)	Softening Point (°C)
70# base asphalt			
Properties	64.1	78.8	50.7
Requirement	60–80	≥40	≥43
SBS modified asphalt			
Properties	57	28.5	85
Requirement	40–60	≥20	≥60

* The test temperature of ductility for 70# base asphalt and SBS modified asphalt was 15 °C and 5 °C, respectively.

**Table 2:** Mixing content of warm-mixing agents.

Additives	Mixing Content
**A**	3 wt.% to the aggregate
**B**	3 wt.% to the bitumen
**C**	6 wt.% to the bitumen
**D**	6 wt.% to the bitumen
**E**	10 wt.% to the bitumen

**Table 3:** Density indexes of aggregates.

Aggregate Type	Limestone	Basalt	Granite
**Bulk Density/g·cm^−3^**	2.692	2.815	2.721
**Apparent Density/g·cm^−3^**	2.720	2.933	2.784

**Table 4:** Moisture content of limestone at different drying times.

Drying Time	0	0.5	1.0	1.5	2.0	2.5	3.0	3.5	4.0	4.5
Moisture content	4.8	3.8	2.9	2.1	1.5	0.9	0.4	0.1	0.01	0

**Table 5:** The surface energy parameters of five liquids.

Liquid Type	γ (mJ/m^2^)	γ^LW^ (mJ/m^2^)	γ^−^ (mJ/m^2^)	γ^+^ (mJ/m^2^)
Distilled water	72.8	21.8	25.5	25.5
Glycol	48.0	29.0	47.0	1.92
Glycerin	64.0	34.0	57.4	3.92
Formamide	58.0	39.0	39.6	2.28
Diiodomethane	50.8	50.8	0	0

**Table 6:** SFE parameters.

Items	γ (mJ/m^2^)	γ^LW^ (mJ/m^2^)	γ^−^ (mJ/m^2^)	γ^+^ (mJ/m^2^)
Base asphalt	34.84	33.26	l.04	0.60
Agent A modified asphalt	43.04	41.04	0.69	1.45
Agent B modified asphalt	33.03	32.98	0.01	0.05
Agent C modified asphalt	34.84	32.59	2.00	0.64
Agent D modified asphalt	37.21	35.77	0.74	0.70
Agent E modified asphalt	34.60	33.32	0.85	0.48
SBS modified asphalt	33.70	31.28	2.60	0.57
Agent A modified SBS	35.06	33.15	2.31	0.39
Agent B modified SBS	34.34	32.13	2.60	0.47
Agent C modified SBS	39.68	34.61	5.96	1.08
Agent D modified SBS	32.29	31.10	1.39	0.25
Agent E modified SBS	37.11	34.11	2.95	0.76
Basalt	42.8	36.9	18.04	0.48
Granite	40.57	35.49	14.66	0.44
Limestone	49.68	40.40	24.85	0.87
Agent E	30.09	25.05	0.67	9.45

**Table 7:** TSR test results for different aggregates.

Aggregate	Basalt	Granite	Limestone
**TSR/%**	80.1	76.9	81.4

**Table 8:** Values of $W_{\mathrm{as}}$ and $W_{\mathrm{asw}}$.

Asphalt Type	$W_{\mathrm{as}}$ (mJ/m^2^)			$W_{\mathrm{asw}}$ (mJ/m^2^)		
**Agent A** **modified asphalt**	Basalt	Granite	Limestone	Basalt	Granite	Limestone
	89.210	86.652	94.992	47.819	50.880	41.100

**Table 9:** Adhesion indicators of asphalts and limestone.

Asphalt Type	$W_{\mathrm{as}}$ (mJ/m^2^)	$W_{\mathrm{asw}}$ (mJ/m^2^)	ER
Base asphalt	82.938	37.442	0.4385
Agent A modified asphalt	94.992	41.100	0.3142
Agent B modified asphalt	82.007	38.276	0.4015
Agent C modified asphalt	75.420	45.005	0.2102
Agent D modified asphalt	83.185	33.994	0.5297
Agent E modified asphalt	85.975	39.468	0.3657

**Table 10:** TSR of WMA using different warm-mixing agents.

Agent	HMA	Agent A	Agent D	Agent E
TSR/%	91.9	81.3	86.6	81.5

**Table 11:** TSR of mixtures with different asphalts and warm-mix additive C.

Asphalt Type	Mixture Type	TSR/%
**SBS** modified asphalt	HMA	91.0
	WMA-C	93.4
**70#** base asphalt	HMA	76.7
	WMA-C	68.9

**Table 12:** TSR values of different mixing methods.

Mixing Method	VV/%	S_T1_/MPa	S_T2_/MPa	TSR/%
**Dry**	5.44	0.717	0.966	81.7
**Wet**	5.47	0.567	0.850	74.3

## Data Availability

All the data are available in the article.
